# Impact of long-term glucose variability on coronary atherosclerosis progression in patients with type 2 diabetes: a 2.3 year follow-up study

**DOI:** 10.1186/s12933-020-01126-0

**Published:** 2020-09-25

**Authors:** Suhua Li, Xixiang Tang, Yanting Luo, Bingyuan Wu, Zhuoshan Huang, Zexiong Li, Long Peng, Yesheng Ling, Jieming Zhu, Junlin Zhong, Jinlai Liu, Yanming Chen

**Affiliations:** 1 Department of Cardiovascular Medicine, The Third Affiliated Hospital of Sun Yat-Sen University, Guangzhou, 510630 China; 2 Department of Endocrinology & Metabolism, Guangdong Provincial Key Laboratory of Diabetology, The Third Affiliated Hospital of Sun Yat-Sen University, Guangzhou, 510630 China; 3 VIP Medical Service Center, The Third Affiliated Hospital of Sun Yat-Sen University, Guangzhou, 510630 China; 4 Department of Ultrasonography, The Third Affiliated Hospital of Sun Yat-Sen University, Guangzhou, 510630 China

**Keywords:** Type 2 diabetes, Glycemic variability, Coronary computed tomography angiography, Atherosclerosis progression

## Abstract

**Background:**

Glycemic variability (GV) confers a risk of cardiovascular events. In this study, we aimed to investigate whether long-term GV has an impact on coronary atherosclerosis progression in patients with type 2 diabetes mellitus (T2DM).

**Methods:**

A total of 396 patients with T2DM who had coronary computed tomography angiography and laboratory data available at baseline and for follow-up evaluations [median 2.3 (1.8–3.1) years] were included. Fasting plasma glucose (FPG) was measured every 1–3 months, and HbA1c was measured quarterly. The coefficient of variation (CV) of HbA1c and FPG were calculated as measures of GV. Quantitative assessment of coronary plaques was performed by measuring the annual change and progression rate of total plaque volume (TPV). Significant progression was defined as annual TPV progression ≥ 15%. Multivariable regression analyses were used to assess the effects of GV on atherosclerosis progression.

**Results:**

In the 396 patients, the annual change in TPV was 12.35 ± 14.23 mm^3^, and annual progression rate was 13.36 ± 12.69%. There were 143 (36.11%) patients with significant progression, and they had a significantly higher CV-HbA1c (*P* < 0.001) and CV-FPG (*P* < 0.001) than those without significant progression. In multivariable regression analyses, both CV-HbA1c and CV-FPG were independent predictors of annual change in TPV [CV-HbA1c: β = 0.241 (0.019–0.462), *P* = 0.034; CV-FPG_:_ β = 0.265 (0.060–0.465), *P* = 0.012], annual TPV progression [CV-HbA1c: β = 0.214 (0.023–0.405), *P* = 0.029; CV-FPG_:_ β = 0.218 (0.037–0.399), *P* = 0.019], and significant atherosclerosis progression [CV-HbA1c: odds ratio [OR] = 1.367 (1.149–1.650), *P* = 0.010; CV-FPG_:_ OR = 1.321 (1.127–1.634), *P* = 0.013].

**Conclusions:**

Long-term GV is associated with accelerated progression of coronary atherosclerosis independent of conventional risk factors in patients with T2DM.

*Trial registration* ClinicalTrials.gov (NCT02587741), October 27, 2015; retrospectively registered

## Background

Type 2 diabetes mellitus (T2DM) represents a major risk factor for the development of coronary artery disease (CAD) and cardiovascular mortality [1]. Evidence indicates that DM is associated with significantly higher coronary artery total plaque volume, independent of other CAD risk factors [2]. In addition, patients with T2DM have greater coronary plaque progression, and in particular significantly greater progression of plaques associated with adverse outcomes [3]. Risk factors related to plaque progression in patients with T2DM include hypertension [2], male sex, and mean plaque burden > 75% at baseline [3].

Intravascular ultrasound is considered as the gold standard for evaluating coronary plaques. However, it is an invasive examination and thus is limited with respect to clinical follow-up [4]. Coronary computed tomography angiography (CTA) has been extensively studied, and is considered as a non-invasive and convenient tool with acceptable accuracy to quantitatively measure coronary artery plaque volume [5–7].

In patients with T2DM, an abundant of evidence has indicated that glycemic variability (GV), independent of hyperglycemia, is associated with an increased risk of cardiovascular diseases [8], adverse changes of cardiac structure and function [9, 10], ischemic stroke [11], cardiovascular mortality [12], all-cause mortality [13–16], and coronary plaque vulnerability [17–21]. However, little is known about the impact of GV on coronary plaque progression in patients with T2DM.

Therefore, the objective of this study was to examine the association of long-term GV with coronary plaque progression among patients with T2DM, independent of conventional CAD risk factors.

## Methods

### Study population

This was a prospective observational cohort study conducted at the Third Affiliated Hospital of Sun Yat-sen University between January 2013 and December 2019. Consecutive patients with T2DM who underwent 320-slice coronary CTA at baseline and follow-up and had laboratory data from baseline and follow-up evaluations were included. The reasons for these patients receiving coronary CTA including angina-like symptoms, an abnormal echocardiogram, positive stress ECG test, abnormal myocardial perfusion scintigraphy, and/or elevated cardiovascular risk [22]. The period between coronary CTA scans was at least 18 months. T2DM was diagnosed according to the 1999 criteria of the World Health Organization (WHO) [23]. Patients with plaque-free coronary arteries at baseline, a history of percutaneous coronary intervention or coronary bypass surgery, < 10 fasting plasma glucose (FPG) measurements or < 5 HbA1c measurements, or inadequate imaging quality for analysis were excluded. Ethical approval was obtained from the Third Affiliated Hospital of Sun Yat-sen University Network Ethics Committee. Informed consent was obtained from all participants.

### Data collection and follow-up

A detailed medical history was obtained from all patients using a standardized questionnaire. Data collected included age, sex, duration of diabetes, comorbidities (i.e., hypertension, hyperlipidemia, and malignancy), lifestyle habits (smoking status and alcohol consumption), the use of medications (i.e., antihypertensive agents, anti-platelet drugs, and statins), current hypoglycemic treatments (i.e., oral hypoglycemic drugs, insulin injections), and hypoglycemic events during the year prior to the collection of baseline data. Body height and weight, systolic blood pressure (SBP) and diastolic blood pressure (DBP), and laboratory studies were obtained at baseline. The laboratory tests included blood urea nitrogen (BUN), creatinine, uric acid (UA), triglycerides, total cholesterol, high-density lipoprotein cholesterol (HDL-C), and low-density lipoprotein cholesterol (LDL-C). Blood was collected after an 8-h overnight fast, and analyzed with a HITACHI 7180 automatic-analyzer. FPG and 2-h postprandial blood glucose (2h-PBG) were measured every 1–3 months, depending on the degree of glycemic control, using the glucose oxidase method. HbA1c was measured quarterly by the D-10 Hemoglobin Testing Program (Bio-Rad) with high-performance liquid chromatography (HPLC). Coronary CTA (320-slice) was performed with an Aquilion ONE CT scanner (Toshiba Medical Systems, Ottawara, Japan) to assess coronary atherosclerosis at baseline and at the final follow-up visit. Anti-hyperglycemic treatments and lifestyle interventions were evaluated by specialist physicians during follow-up visits, and changes were based on glucose control status.

### Assessment of glycemic variability

Participants with at least 10 FPG and 5 HbA1c measurements during the study were included in the analyses of GV. For each participant, the intra-personal mean and standard deviation (SD) of all recorded FPG and HbA1c measurements were calculated. The coefficient of variation (CV) was defined as the ratio of the SD over the mean FPG or mean HbA1c, and the CV-HbA1c and CV-FPG were considered measures of GV. Considering that the frequency of follow-up visits could influence the calculation results, the CV-HbA1c and CV-FPG were further adjusted by dividing by the square root of the ratio of total visits divided by total visits minus 1 [9–11].

### Acquisition and analysis of coronary CTA images

The 320-slice coronary CTA was performed according to a predefined standard operating procedure to ensure optimal image quality. An oral dose (50–100 mg) of metoprolol (Astrazeneca, Wuxi, China) was given to patients with a resting heart rate > 70 bpm. Isovue-370 (50–100 ml, Bracco Diagnostics, Guangzhou, China) was injected intravenously at a flow rate of 6.0 ml/s, followed by a 20 ml saline flush at a flow rate of 4.0 ml/s. Coronary CTA was performed with 0.5-mm detector element, gantry rotation time of 350 ms, and up to 16 cm of coverage in the Z direction. Tube voltage was 100–135 kV, and the maximal tube current was 400–580 mA. The follow-up scan was performed at least 18 months after the initial CTA using the same protocol.

Datasets were transferred to a workstation for quantitative assessment of coronary plaques using semi-automated plaque analysis software. All images were analyzed by 2 experienced radiologists with more than 4 years of experience in evaluating cardiac CT who were blinded to patient clinical data. Coronary arteries were assessed according to the modified 17-segment American Heart Association classification [24, 25]. Baseline and follow-up coronary lesions were matched using branch points as landmarks [25]. The lumen and vessel borders were automatically detected by the software in the 3-dimensional reconstructed images, and then manually corrected by the radiologists. Total plaque volume (TPV) based on a per-patient analysis was calculated by a calculus sum of the cross-sectional area of the vessel border minus the cross-sectional area of the lumen. The annual change and progression rate of TPV were calculated. Clinically significant atherosclerosis progression was defined as an annual TPV progression rate ≥ 15% [26]. The percentage of patients with significant progression was calculated.

### Statistical analysis

Database management and statistical analysis was performed by using SPSS version 22.0 software for Windows (SPPS Inc., Chicago, IL, USA). Descriptive statistics were presented as mean ± standard deviation for continuous variables, or as number and percentage for categorical variables. Pearson chi-square tests for trend, and Spearman rank correlation tests were used to examine relations between coronary artery plaque parameters and GV. Univariate linear regression analysis was performed to assess the non-adjusted relations between GV and the annual change and progression rate of TPV, and to evaluated the association between GV and clinically significant atherosclerosis progression. Multivariate regression models were performed to adjust for confounding factors, including age, sex, body mass index (BMI), diabetes duration, smoking status, alcohol consumption, hypertension, hypoglycemia rate, triglycerides, LDL-C, HDL-C, FPG, 2h-FBG, HbA1c, estimated glomerular filtration rate (eGFR), UA, Homeostatic Model Assessment of Insulin Resistance (HOMA-IR), urine albumin-to-creatinine ratio (UACR), and medications. A 2-tailed *P* value < 0.05 was considered to indicate statistical significance.

## Results

### Patient selection

**Fig. 1:**
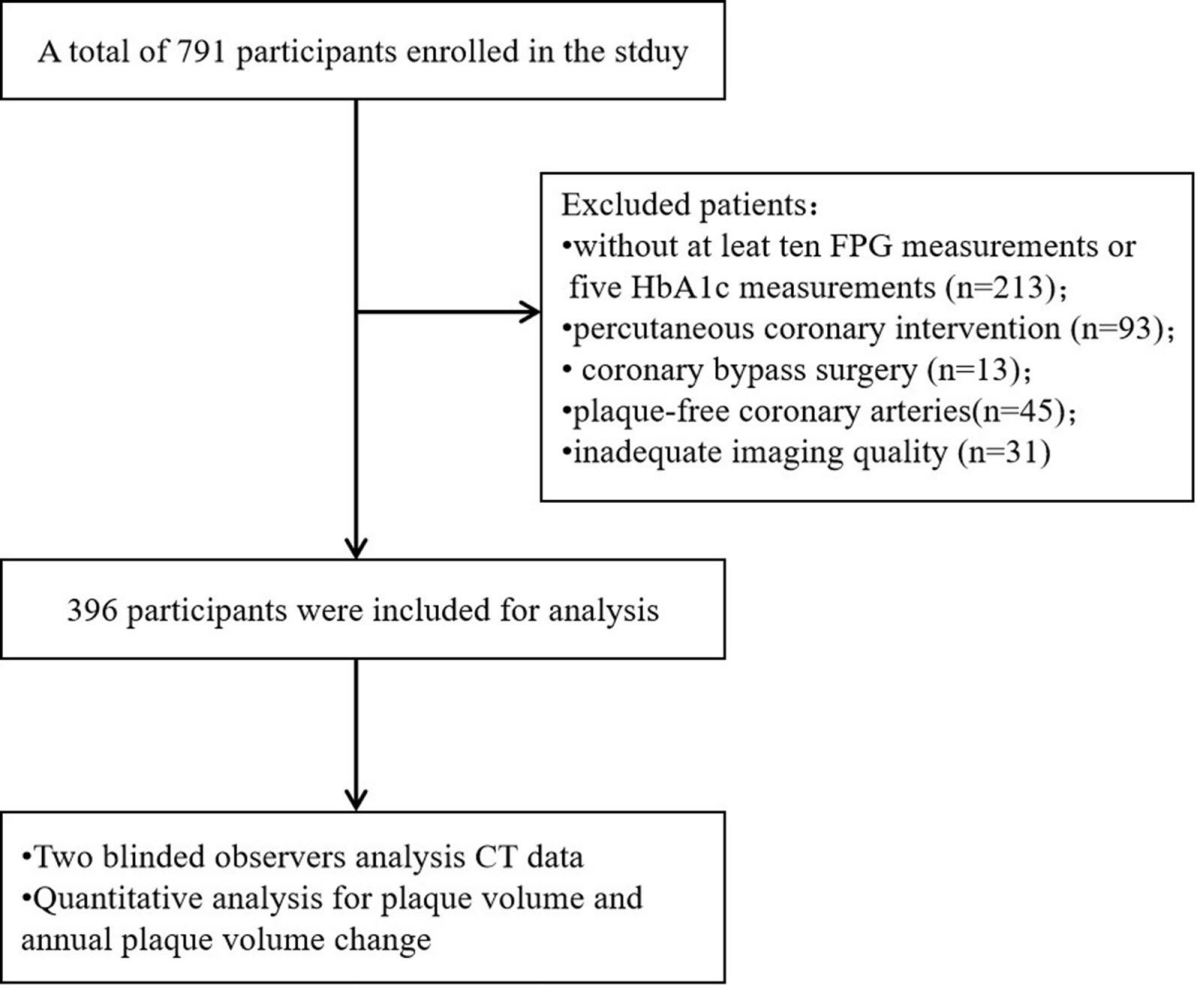
Flowchat of the present study

In this cohort study, a total of 791 consecutive patients who had paired coronary CTA and laboratory data at both baseline and follow-up evaluations were enrolled. Participants with plaque-free coronary arteries (n = 45), < 10 FPG measurements or < 5 HbA1c measurements (n = 213), a history of percutaneous coronary intervention (n = 93) or coronary bypass surgery (n = 13), and inadequate imaging quality for analysis (n = 31) were excluded. Finally, a total of 396 patients (200 males, 196 females; mean age 46.30 ± 10.9 years) with 1577 coronary segments with visible plaques were included in the analysis (Fig. 1). The median diabetes duration was 4.0 (interquartile range [IQR]: 2.0–8.0) years at baseline, and the median follow-up duration was 2.3 (IQR: 1.8–3.1) years. The mean baseline HbA1c was 6.7 ± 1.4%, and FPG was 6.9 ± 2.3 mmol/L.

### Total plaque volume at baseline and follow-up

**Fig. 2:**
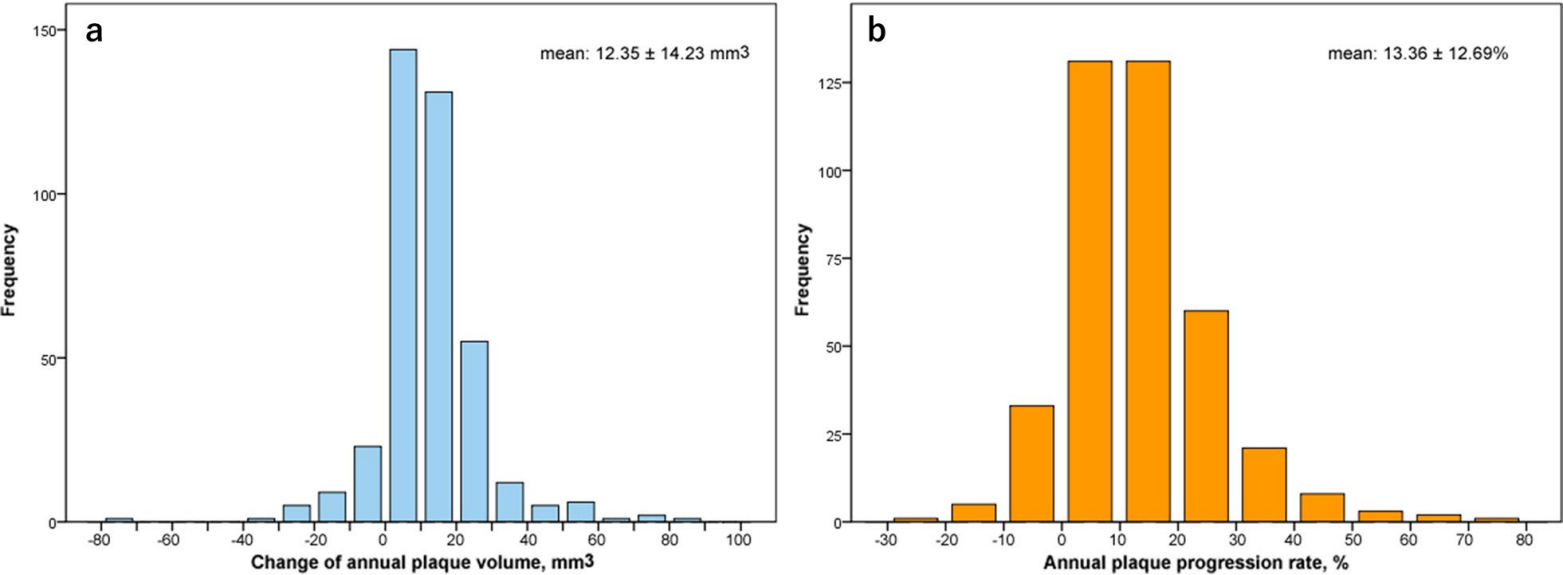
Distribution of the annual change and progression rate in TPV

**Fig. 3:**
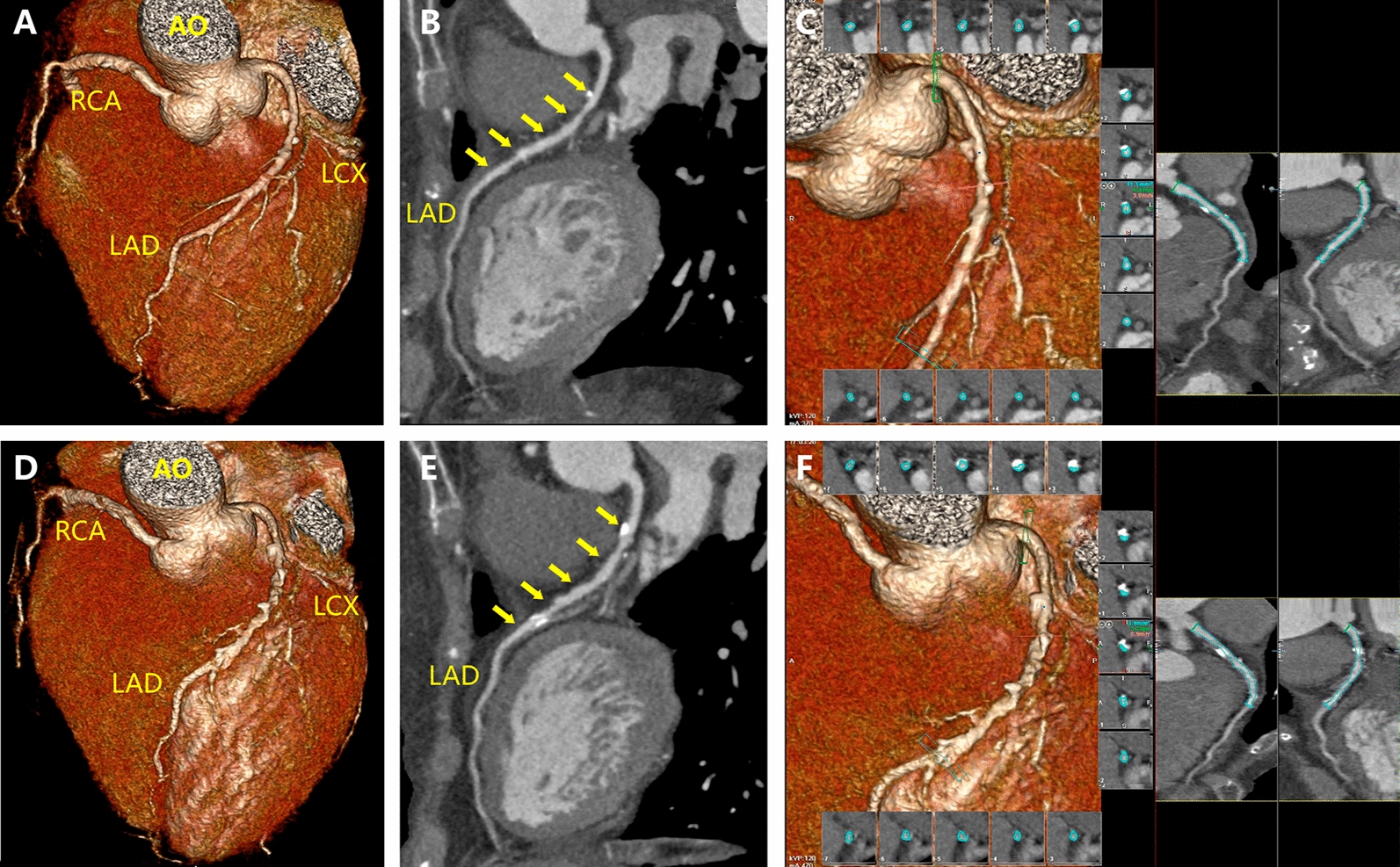
Representative imaging for the change of the total plaque volume at the beginning and the follow-up. The 3-dimensional reconstruction and analysis of coronary CTA images of a 60-year-old female patient with type 2 diabetes were performed. The baseline CTA showed a moderate stenosis in the proximal segment of the left anterior descending coronary artery (**a**, **b**), with a total plaque volume of 119 mm^3^ (**c**). After a 2.8-year follow up, clinically significant progression (**d**–**f**) was observed, with the total plaque volume progressed to 194 mm^3^. The annual progression rate in total plaque volume was 22.51%

The initial TPV of all participants was 108.60 ± 42.61 mm^3^ based on a per-patients analysis. The absolute change in TPV was 28.34 ± 33.72 mm^3^ after a median follow-up of 2.3 (IQR: 1.8–3.1) years. Thus, the mean annual change in TPV was 12.35 ± 14.23 mm^3^, and the mean annual TPV progression rate was 13.36 ± 12.69% (Fig. 2). There were 143 (36.11%) patients with clinically significant atherosclerosis progression (annual TPV progression rate ≥ 15%), while the other 253 (63.9%) patients did not have significant progression. Representative imaging studies showing progression in total plaque volume were shown in Fig. 3.

**Fig. 4:**
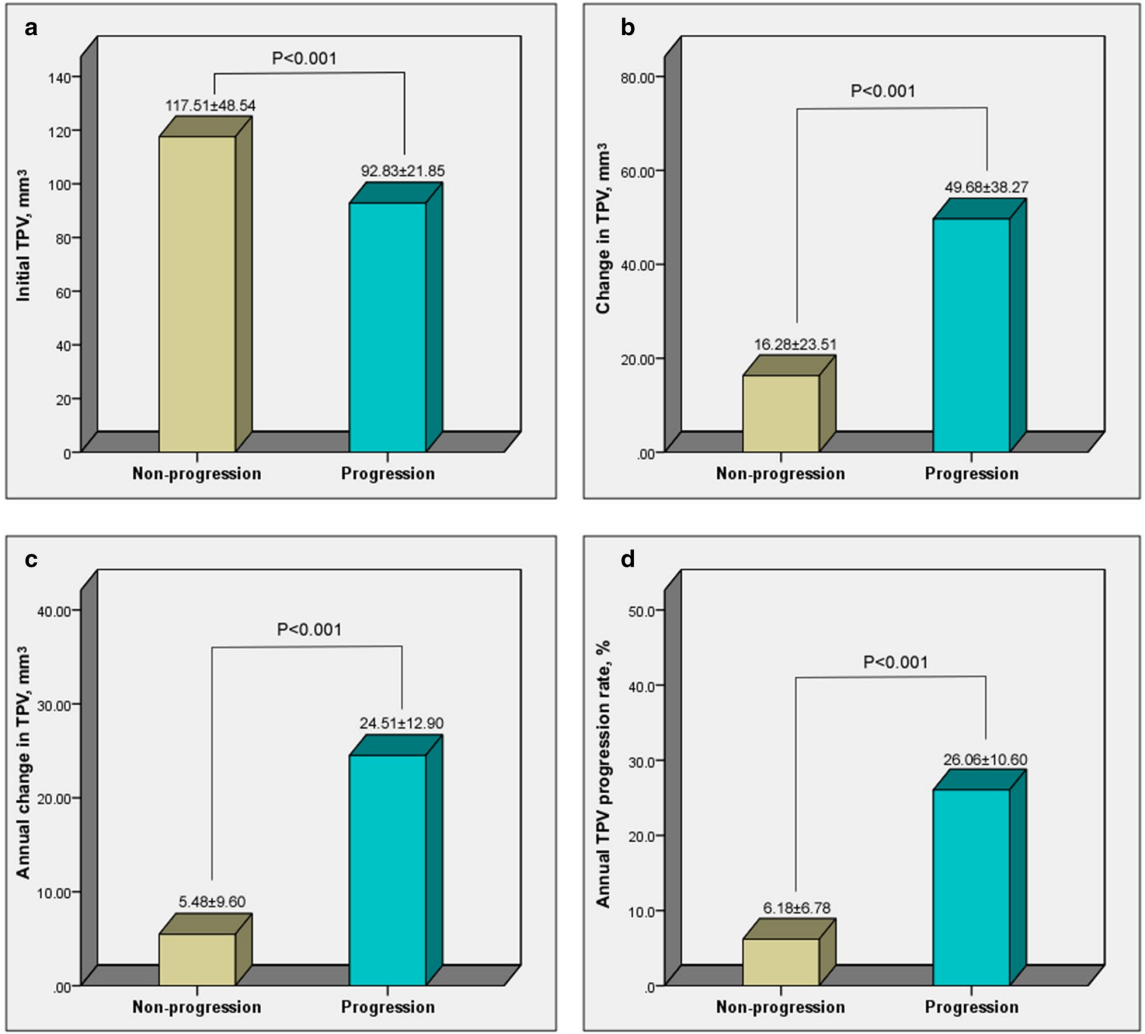
Comparison between patients with non-progression and progression in coronary atherosclerosis

Patient baseline sociodemographic and clinical data were summarized in Table 1. Compared to patients without atherosclerosis progression, HbA1c, FPG, 2h-PBG, HOMA-IR, total cholesterol, and LDL-C were higher in patients with progression (all, *P* < 0.05), while the percentages of patients who consumed alcohol, used anti-platelet medications, and took statins were lower (all, *P* < 0.05). Although the initial TPV was lower in patients with progression, after follow-up the absolute change in TPV (*P* < 0.001), annual change in TPV (*P* < 0.001), and annual TPV progression rate (*P* < 0.001) were significantly higher than in patients without progression (Fig. 4).

**Table 1:** Baseline characteristics of patients with or without clinically significant atherosclerosis progression

Variables	Total	Non-progression (n = 253)	Progression (n = 143)	*P*-value
Age, years	63.0 ± 10.9	63.5 ± 10.3	62.1 ± 11.9	0.222
Male, n (%)	200 (50.5)	126 (49.8)	74 (51.7)	0.659
BMI, kg/m^2^	26.4 ± 5.5	26.4 ± 5.4	26.5 ± 5.6	0.798
Smoking, *n* (%)	107 (27.0)	65 (25.7)	42 (29.4)	0.328
Alcohol, n (%)	51 (12.9)	40 (15.8)	11 (7.7)	0.021
Hyperlipidemia, n (%)	121 (30.6)	73 (28.9)	48 (33.6)	0.328
Hypertension, *n* (%)	266 (67.2)	177 (70.0)	89 (62.2)	0.116
SBP, mmHg	140.0 ± 18.8	140.5 ± 19.6	138.9 ± 16.9	0.380
DBP, mmHg	79.3 ± 11.6	79.7 ± 11.7	78.6 ± 11.5	0.394
HR, bpm	76.9 ± 12.2	77.0 ± 12.7	76.5 ± 11.4	0.694
Diabetes-related variables				
Diabetes duration, years	4.0 (2.0–8.0)	4.3 (2.0–9.0)	3.5 (2.0–6.0)	0.125
HbA1c, %	6.7 ± 1.4	6.5 ± 1.1	7.1 ± 1.7	< 0.001
Number of HbA1c measurements, n	7.5 ± 1.8	7.4 ± 1.8	7.7 ± 1.9	0.436
Time interval between HbA1c(s), months	4.0 (3.5–4.6)	4.0 (3.8–4.5)	3.9 (3.4–4.7)	0.384
CV-HbA1c, %	16.73 ± 7.87	15.48 ± 6.17	18.96 ± 9.83	< 0.001
FPG, mmol/L	6.9 ± 2.3	6.57 ± 1.72	7.49 ± 3.06	0.001
Number of FPG measurements, n	9.9 ± 2.0	9.8 ± 1.9	10.1 ± 2.3	0.226
Time interval between FPGs, months	3.0 (2.6–3.5)	3.1 (2.7–3.6)	2.9 (2.4–3.3)	0.363
CV-FPG, %	13.80 ± 10.20	12.08 ± 8.47	16.84 ± 12.14	< 0.001
2h-PBG, mmol/L	9.65 ± 2.40	9.20 ± 1.74	10.44 ± 3.10	< 0.001
FCP, nmol/L	1.83 ± 1.26	1.83 ± 1.24	1.82 ± 1.30	0.933
2h-PCP, nmol/L	4.63 ± 2.48	4.69 ± 2.54	4.53 ± 2.36	0.536
Fasting insulin, mU/L	79.21 ± 50.43	77.35 ± 50.01	82.51 ± 51.16	0.328
2h-insulin, mU/L	220.99 ± 141.71	226.71 ± 139.48	210.86 ± 145.52	0.285
HOMA-IR	24.21 ± 19.12	22.27 ± 15.59	27.65 ± 23.81	0.016
UACR, mg/g	1.65 (0.85–3.72)	1.46 (0.79–4.18)	1.76 (0.85–3.02)	0.921
Hypoglycemia rates, %	26 (6.6)	17 (6.7)	9 (6.3)	0.870
Other laboratory tests				
TC, mmol/L	4.74 ± 1.13	4.60 ± 1.18	5.00 ± 0.99	0.001
TG, mmol/L	1.49 (1.00–2.15)	1.49 (1.02–2.20)	1.50 (0.94–2.11)	0.637
HDL-C, mmol/L	1.11 ± 0.28	1.10 ± 0.26	1.12 ± 0.31	0.162
LDL-C, mmol/L	2.95 ± 0.93	2.83 ± 0.94	3.15 ± 0.86	0.001
apoA1, g/L	1.35 ± 0.29	1.34 ± 0.32	1.38 ± 0.22	0.084
apoB100, g/L	1.12 ± 0.42	1.09 ± 0.42	1.16 ± 0.41	0.088
LP_a, mg/L	193.3 ± 246.0	194.2 ± 254.0	191.52 ± 232.0	0.916
Cr, µmol/L	76.7 ± 20.7	77.0 ± 21.6	76.2 ± 19.0	0.701
eGFR, mL/min/1.73 m^2^	83.1 ± 19.0	82.2 ± 19.7	84.8 ± 17.8	0.205
BUN, µmol/L	5.64 ± 1.71	5.64 ± 1.69	5.64 ± 1.74	0.972
UA, µmol/L	379.0 ± 108.0	380.0 ± 102.3	377.2 ± 117.8	0.808
Medications				
Insulin, n (%)	83 (21.0)	52 (20.6)	31 (21.7)	0.807
Metformin, n (%)	234 (59.0)	146 (57.8)	88 (62.6)	0.706
Glucosidase inhibitor, n (%)	84 (21.2)	45 (17.8)	39 (27.3)	0.028
Sulfonylureas, n (%)	44 (11.1)	29 (11.5)	15 (10.5)	0.757
DPP-4 inhibitors/GLP-1R, n (%)	34 (8.6)	27 (10.7)	7 (4.9)	0.048
Glinides, n (%)	7 (1.8)	6 (2.4)	1 (0.7)	0.223
Anti-platelet, n (%)	285 (72.0)	196 (77.5)	89 (62.2)	0.001
Statin, n (%)	295 (74.5)	197 (77.9)	98 (68.5)	0.041
ACEI/ARB, n (%)	179 (45.2)	121 (47.8)	58 (40.6)	0.163
β-blocker, n (%)	122 (30.8)	85 (33.6)	37 (25.9)	0.110
CCB, n (%)	130 (32.8)	90 (35.6)	40 (28.0)	0.122
Diuretic, n (%)	58 (14.6)	42 (16.6)	16 (11.2)	0.143

Data are mean (SD), median (25th to 75th percentile) or n (%)

BMI, body mass index; SBP, systolic blood pressure; DBP, diastolic blood pressure; HR, heart rate; CV, coefficient of variation; FPG, fasting plasma glucose; PBG, postprandial blood glucose; FCP, Fasting C-peptide; 2h-PCP, 2hour-postprandial C-peptide; HOMA-IR, homeostatic model assessment of insulin resistance; UACR, urine albumin-to-creatinine ratio; Cr, plasma creatinine; BUN, blood urea nitrogen; UA, uric acid; TC, total cholesterol; TG, triglycerides; HDL-C, high density lipoprotein cholesterol; LDL-C, low density lipoprotein cholesterol; ACEI/ARB, angiotensin converting enzyme inhibitor/angiotensin receptor blocker; CCB, calcium channel blockers

### Glycemic variability

**Fig. 5:**
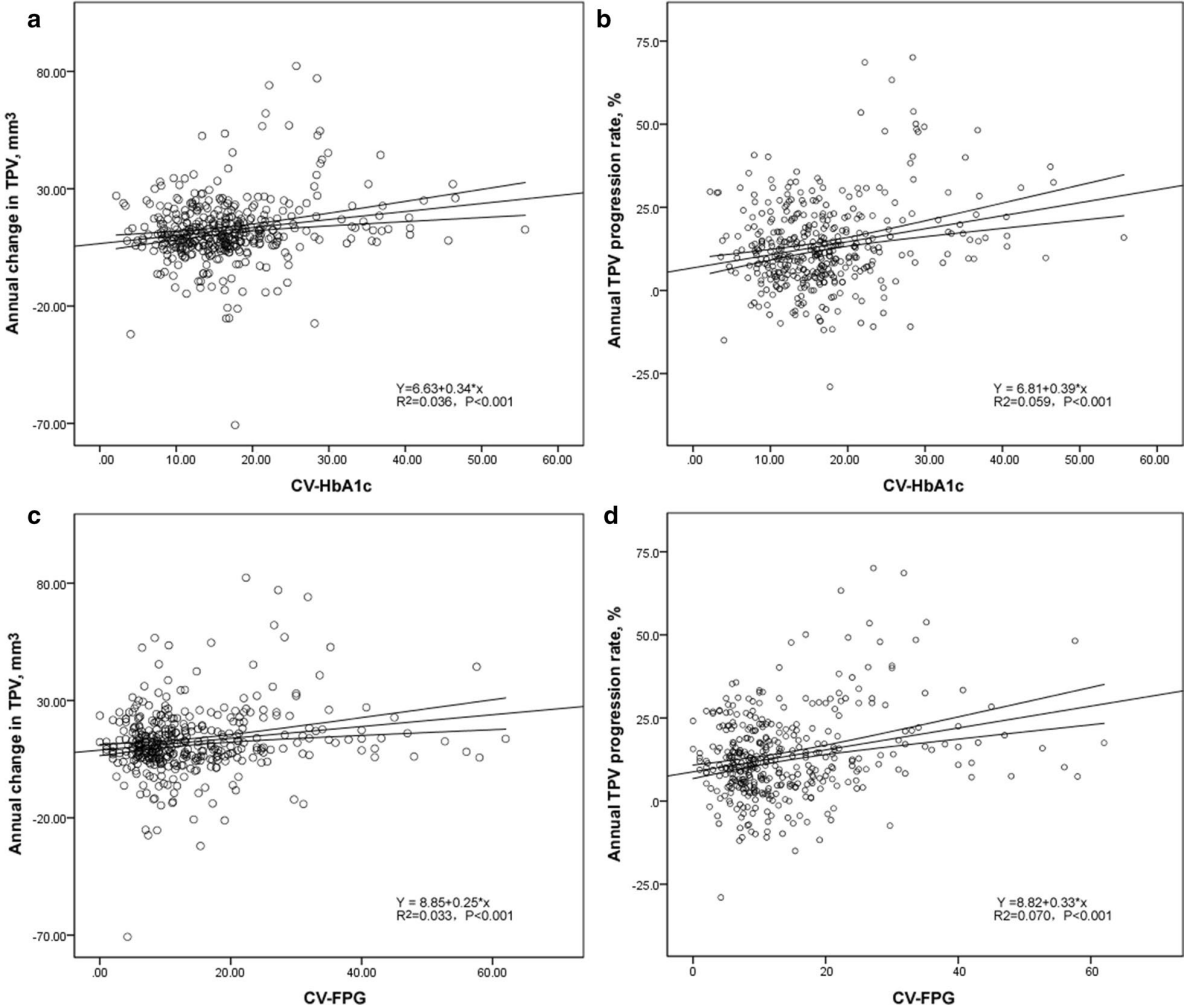
Correlation between glucose variability and atherosclerosis progression. **a** Y = 6.63 + 0.34 * x, R^2^ = 0.036, *P* < 0.001. **b** Y = 6.81 + 0.39 * x, R^2^ = 0.059, *P* < 0.001. **c** Y = 8.85 + 0.25 * x, R^2^ = 0.033, *P* < 0.001. **d** Y = 8.82 + 0.33 * x, R^2^ = 0.070, *P* < 0.001

The mean CV-HbA1c was 16.73 ± 7.87% in all patients, and the mean CV-FPG was 13.80 ± 10.20%. When compared to patients without atherosclerosis progression, patients with progression have markedly higher GV, as indicated by CV-HbA1c (18.96 ± 9.83% vs. 15.48 ± 6.17, *P* < 0.001) and CV-FPG (16.84 ± 12.14 vs. 12.08 ± 8.47, *P* < 0.001). As shown in Fig. 5, both CV-HbA1c and CV-FPG were positively correlated with TPV annual change and progression rate (all, *P* < 0.001).

### Linear regression analysis assessing the effect of glycemic variability on atherosclerosis progression

In the univariate analysis, potential factors associated with TPV annual change and progression rate were HbA1C, CV-HbA1C, FPG, CV-FPG, 2h-FBG, LDL-C, HOMA-IR, use of anti-platelet drugs, use of statins, and alcohol consumption (Table 2). In the multivariate regression analysis adjusted for age, sex, BMI, diabetes duration, smoking, alcohol consumption, hypertension, hypoglycemia rate, triglycerides, LDL-C, HDL-C, FPG, 2h-FBG, HbA1c, eGFR, UA, HOMA-IR, UACR, and medications, elevated indices of GV, both CV-HbA1C and CV-FPG, remained strong determinants of annual change in TPV [CV-HbA1C: β (95% CI) = 0.241 (0.019–0.462), *P* = 0.034, and CV-FPG: β (95% CI) = 0.265 (0.060–0.465), *P* = 0.012] and annual TPV progression rate [CV-HbA1C: β (95% CI) = 0.214 (0.023–0.405), *P* = 0.029, and CV-FPG: β (95% CI) = 0.218 (0.037–0.399), *P* = 0.019] (Table 2).

**Table 2:** Linear regression analysis assessing the effects of potential risk factors on plaque volume progression

Variables^a^	Univariate			Multivariate^b^		
	Sβ	95%CI	P-value	Sβ	95%CI	*P*-value
Annual change in TPV						
CV-HbA1C	0.242	0.062, 0.438	0.004	0.241	0.019, 0.462	0.034
HbA1C	0.250	0.154, 0.346	< 0.001	0.275	0.073, 0.477	0.009
CV-FPG	0.233	0.076, 0.420	0.003	0.265	0.060, 0.465	0.012
FPG	0.245	0.149, 0.341	< 0.001	0.270	0.041, 0.499	0.022
2h-PBG	0.299	0.204, 0.394	< 0.001	0.251	0.022, 0.760	0.051
LDL-C	0.236	0.140, 0.332	< 0.001	0.243	0.089, 0.562	0.023
HOMA-IR	0.194	0.036, 0.292	< 0.001	− 0.046	− 0.264, 0.172	0.671
Anti-platelet	− 0.156	− 0.254, − 0.058	0.002	− 0.281	− 0.525, − 0.037	0.025
Statin	− 0.130	− 0.228, − 0.032	0.010	− 0.278	− 0.508, − 0.049	0.019
Alcohol	− 0.116	− 0.214, − 0.012	0.021	− 0.193	− 0.386, 0.001	0.052
Annual TPV progression rate						
CV-HbA1C	0.243	0.078, 0.439	0.006	0.214	0.023, 0.405	0.029
HbA1C	0.282	0.187, 0.377	< 0.001	0.362	0.132, 0.591	0.003
CV-FPG	0.265	0.069, 0.460	0.004	0.218	0.037, 0.399	0.019
FPG	0.257	0.161, 0.352	< 0.001	0.253	0.039, 0.467	0.022
2h-FBG	0.316	0.221, 0.410	< 0.001	0.215	0.024, 0.665	0.055
LDL-C	0.230	0.133, 0.326	< 0.001	0.295	0.106, 0.484	0.003
HOMA-IR	0.205	0.108, 0.303	< 0.001	− 0.122	− 0.324, 0.080	0.231
Anti-platelet	− 0.170	− 0.267, − 0.073	0.001	− 0.311	− 0.540, − 0.082	0.009
Statin	− 0.148	− 0.246, − 0.050	0.003	− 0.383	− 0.614, − 0.152	0.002
Alcohol	− 0.116	− 0.215, − 0.017	0.021	− 0.201	− 0.407, − 0.041	0.058

CV, coefficient of variation; FPG, fasting plasma glucose; PBG, postprandial blood glucose; HOMA-IR, homeostatic model assessment of insulin resistance; LDL-C, low density lipoprotein cholesterol

^a^The variables which got a level of *P* < 0.05 in the univariate analysis was presented in this table

^b^Multivariate regression analysis was adjusted for age, sex, BMI, diabetes duration, smoking, alcohol consumption, hypertension, hypoglycemia rate, triglyceride, LDL-C, HDL-C, FPG, 2h-FBG, HbA1c, eGFR, UA, HOMA-IR, UACR, and medications

### Logistic regression analysis assessing the value of GV for predicting clinically significant atherosclerosis progression

In the univariate analysis, potential factors associated with clinically significant atherosclerosis progression included HbA1C, CV-HbA1C, FPG, CV-FPG, 2h-FBG, LDL-C, HOMA-IR, use of anti-platelet drugs, use of statins, use of glucosidase inhibitors, and alcohol consumption (Table 3). In the multivariate analysis adjusting for age, sex, BMI, diabetes duration, smoking, alcohol consumption, hypertension, hypoglycemia rate, triglycerides, LDL-C, HDL-C, FPG, 2h-FBG, HbA1c, eGFR, UA, HOMA-IR, UACR, and medications, for each 1 unit increase in CV-HbA1c, the risk of clinically significant atherosclerosis progression increased by 36.7% [OR (95% CI) = 1.367 (1.149–1.650), *P* = 0.010], and for each 1 unit increase in CV-FPG, the risk increased by 32.1% [OR (95%CI) = 1.321 (1.127–1.634), *P* = 0.013].

**Table 3:** Logistic regression analysis assessing the value of potential risk factors for predicting clinically significant atherosclerosis progression

Variables^a^	Univariate			Multivariate^b^		
	OR	95%CI	P-value	OR	95%CI	*P*-value
CV- HbA1C	1.458	1.230, 1.688	< 0.001	1.367	1.149, 1.650	0.010
HbA1C	1.675	1.483, 1.897	< 0.001	1.499	1.208, 1.725	< 0.001
CV-FPG	1.445	1.123, 1.767	0.002	1.321	1.127, 1.634	0.013
FPG	1.398	1.284, 1.525	< 0.001	1.301	1.184, 1.532	0.018
2h-PBG	1.281	1.152, 1.424	< 0.001	1.465	0.889, 3.133	0.060
LDL-C	1.461	1.164, 1.835	0.001	1.378	1.078, 1.763	0.011
HOMA-IR	1.015	1.004, 1.026	0.010	1.010	0.996, 1.023	0.171
Anti-platelet	0.479	0.306, 0.751	0.001	0.426	0.246, 0.737	0.002
Statin	0.619	0.390, 0.982	0.042	0.608	0.412, 0.807	0.036
Glucosidase inhibitor	1.725	1.058, 2.814	0.029	1.601	0.140, 6.221	0.348
Alcohol	0.444	0.220, 0.895	0.023	0.476	0.223, 1.014	0.054

CV, coefficient of variation; FPG, fasting plasma glucose; PBG, postprandial blood glucose; HOMA-IR, homeostatic model assessment of insulin resistance; LDL-C, low density lipoprotein cholesterol

^a^The variables which got a level of *P* < 0.05 in the univariate analysis was presented in this table

^b^Multivariate regression analysis was adjusted for age, sex, BMI, diabetes duration, smoking, alcohol consumption, hypertension, hypoglycemia rate, triglyceride, LDL-C, HDL-C, FPG, 2h-FBG, HbA1c, eGFR, UA, HOMA-IR, UACR, and medications

## Discussion

The present study revealed that increased long-term GV was significantly associated with coronary artery plaque progression, independent of HbA1C, FPG, and the other conventional CAD risk factors.

It has been shown that the overall coronary plaque burden is a good predictor of future cardiovascular events [27]. In this study, we use coronary CTA to quantitatively assess the progression of coronary atherosclerosis [28–30]. The method has been validated with respect to plaque volume in relation to histological examination and intravascular ultrasound [4, 31].

### Coronary atherosclerosis progression in T2DM

Abundant studies have shown that T2DM influences the development and progression of CAD, and is related to the elevated risk of cardiovascular events and mortality. DM is independently related to higher TPV, leading to subsequent adverse cardiac events [2]. Moreover, previous studies have indicated that DM has an incremental impact on coronary plaque progression, and associated subsequent cardiac events [32–35]. There is a twofold greater progression in TPV in diabetic patients than non-diabetic patients [32]. In this study, we found that the annual TPV progression rate in patients with T2DM was approximately 13%, and more than one-third of patients demonstrated clinically significant coronary atherosclerosis progression. In addition, our results showed that poor blood glucose control (elevated HbA1c and FPG) were independent risk factors for atherosclerosis progression; a finding consistent with results reported by Berry et al. [36]. In addition, previous studies showed that male sex, mean plaque burden > 75% at baseline [3], statin use and hypertension [2] were also associated with plaque progression in patients with T2DM. Similarly, our results showed that several conventional risk factors, such as LDL-C level, anti-platelet drug use, and statin use, were independently associated with the annual TPV progression rate and clinically significant atherosclerosis progression. Different from previous studies, we showed that increased CV-HbA1C and CV-FPG were indicators of coronary plaque progression, independent of HbA1c, FPG, and the other conventional risk factors.

### GV and the risk of coronary atherosclerosis progression

It has been shown that a higher GV is accompanied by an unfavorable metabolic profile and is associated with an increased risk of developing complications and mortality in patients with DM [37–39]. Abundant evidence has indicated that increased long-term GV, assessed by long-term fluctuations of HbA1c or FPG, can predict the risk of CVD [14, 40–44] in patients with T2DM, the prognosis of acute lung diseases in patients with DM [45], and malignancies in the general population [46]. Increased GV after transcatheter aortic valve implantation is associated with an increased risk of major complications within 30 days [47], and reducing GV may represent a new therapeutic strategy for preventing the development of heart failure with preserved ejection fraction in patients with T2DM [48]. In addition, combining GV and HbA1c may achieve the highest accuracy for determining thrombotic risk [49]. Furthermore, studies have shown that glucose fluctuations strongly contribute to increased coronary plaque vulnerability at the culprit [17, 18] and non-culprit lesions of acute coronary syndrome [19–21]. Therefore, elevated GV may promote coronary atherosclerosis progression, which is the primary contributor of cardiovascular events. However, there is little data regarding the role of long-term GV in coronary atherosclerosis progression in patients with T2DM and stable CAD. The present study fills the gap in knowledge of the relation between long-term GV and the risk of CVD, which is one of strengths of the present study. The present study provides further evidence that long-term GV is independently associated with coronary plaque progression before the occurrence of clinical cardiovascular events. This may partly explain the finding of our previous studies, which demonstrated that GV was a novel risk factor for both the long-term adverse changes in cardiac function [9, 10] and 10-year risk of CVD in patients with T2DM [8]. Our findings suggest that maintaining long-term glycemic stability for patients with T2DM may delay the progression of CAD and improve clinical outcomes.

The present study explored the role of GV on coronary atherosclerosis progression, and the results may partly explain why GV contributes to adverse cardiovascular events and CVD-related mortality. Of note, it is very important to evaluate the effects of anti-diabetic agents on GV, and their potential to attenuate the progression of coronary atherosclerosis [50, 51]. Recent evidence has indicated that DPP-4 inhibitors can improve GV and stabilize coronary artery plaques to a degree greater than that of usual medical care [52, 53]. Other emerging therapeutic agents, such as SGLT2 inhibitors, have also been shown to effectively reduce glucose fluctuations [54]. However, whether they can delay, or even reverse the progression of atherosclerosis, remains unclear. More studies should be designed to explore the best hypoglycemic therapies for improving GV and decreasing atherosclerosis progression.

### Potential mechanisms by which GV affects coronary atherosclerosis progression

The mechanisms by which increased GV contribute to coronary artery plaque progression in T2DM patients are yet to be elucidated. There are several potential explanations. Important drivers of DM associated with atherosclerosis progression and plaque instability include oxidative stress, inflammation, endothelial dysfunction, and alterations in mineral metabolism [55–58]. Positive correlations among long-term GV (HbA1c-SD) and short-term GV (the mean amplitude of glycemic excursions) with markers of oxidative stress and inflammation in T2DM patients have been reported [59], which suggests that both acute and chronic glucose fluctuations can induce oxidative stress and chronic inflammation. Thus, glucose fluctuations can enhance inflammation and oxidative stress [60, 61], elevate the adhesion of monocytes to endothelial cells, cause cell apoptosis [62], and promote endothelial senescence [63], subsequently leading to cardiovascular injury and dysfunction. In addition, glucose instability strongly predicts an increased incidence of hypoglycemia in patients with DM [64]. The underlying mechanism by which hypoglycemia increases CVD risk may be the release of inflammatory cytokines, increased platelet activation, endothelial dysfunction, and a sympatho-adrenal response that might induce arrhythmias and increase cardiac workload [65–67].

## Limitations

The present study has several limitations. First, it was an observational cohort study performed at a single center, so residual confounders are hard to avoid and the results should be interpreted with some caution. Second, the values and intervals in FPG and HbA1c measurements varied for each subject during the follow-up period, although the effect of the frequency of FPG and HbA1c measurements on variability was adjusted. Third, coronary CTA was performed due to suspicion of CAD according to the clinical judgment of the referring physician. This study enrolled patients that underwent coronary CTA for suspected CVD; it did not include asymptomatic patients with normal electrocardiograms, which may cause a selection bias. Fourth, only quantitative CTA analysis was performed for visually determined plaques at the baseline and follow-up coronary CTA; qualitative analysis of coronary plaques was unavailable. In addition, measurement of plaque volume may be affected by technical factors such as tube voltage and contrast material injection rate.

## Conclusions

The current study demonstrated that long-term GV was associated with accelerated progression of coronary plaques, independent of conventional risk factors. Further studies are warranted to assess the effect of maintaining long-term glucose stability on coronary plaque progression and its relations to clinical outcomes.

## Data Availability

The datasets used and/or analyzed during the current study are available from the corresponding author on reasonable request.

## Abbreviations

CAD: Coronary artery diseases
DM: Diabetes mellitus
T2DM: Type 2 diabetes mellitus
GV: Glycemic variability
CV: Coefficient of variation
FPG: Fasting plasma glucose
WHO: World Health Organization
SBP: Systolic blood pressure
DBP: Diastolic blood pressure
BUN: Blood urea nitrogen
UA: Uric acid
HDL-C: High-density lipoprotein cholesterol
LDL-C: Low-density lipoprotein cholesterol
HPLC: High performance liquid chromatography
SD: Standard deviation
